# Effects of synthetic fertilizer and farm compost on soil nematode community in long-term crop rotation plots: A morphological and metabarcoding approach

**DOI:** 10.1371/journal.pone.0230153

**Published:** 2020-03-17

**Authors:** Gisèle L. Herren, Joos Habraken, Lieven Waeyenberge, Annelies Haegeman, Nicole Viaene, Mathias Cougnon, Dirk Reheul, Hanne Steel, Wim Bert

**Affiliations:** 1 Nematology Research Unit, Department of Biology, Ghent University, Ghent, Belgium; 2 Plant Sciences Unit, ILVO – Flanders Research Institute for Agriculture, Fisheries and Food, Merelbeke, Belgium; 3 Research Unit Plant Breeding and Sustainable Crop Production, Department of Plants and Crops, Faculty of Bioscience and Engineering, Ghent University, Ghent, Belgium; Universidade de Santiago de Compostela, SPAIN

## Abstract

Soil biodiversity plays a key regulation role in the ecosystem services that underpin regenerative sustainable agriculture. It can be impacted by agricultural management techniques, both positively (through measures such as compost application) and negatively (through, for example, application of synthetic nitrogen). As one of the most numerous members of the soil biota, nematodes are well established as indicators for the soil food web. However, compost application also includes the addition of nematodes present in compost and their subsequent survival in soil is unknown. Nematode communities within the compost applied to soil, and nematode communities in the soil of a multi-year rotational cropping field trial in Melle (Belgium) were studied using morphological and metabarcoding techniques. Compost (C) and nitrogen fertilizer (NF) treated plots were compared. Three replicate plots were investigated for each of the following treatments: C application only; C and NF application; NF only; no C and no NF (control). Plots were sampled six times between 2015–2017, before and after C or NF were added each spring and after crop harvest (except for 2017). NF treatment resulted in a significant decrease of fungal feeding and predatory nematodes, while herbivorous nematodes were positively affected. Remarkably, we did not find compost addition to exert any noticeable effects on the soil nematode community. The morphological and metabarcoding data resulted in different results of the nematode community composition. However, trends and patterns in the two data sets were congruent when observed with NMDS plots and using the nematode maturity index. Metabarcoding of individual compost nematode taxa demonstrated that nematodes originating from compost did not persist in soil.

## Introduction

Soil biodiversity is known to play a key role in regulating the delivery of many ecosystem goods and services, including primary production, decomposition, water purification, erosion control, biological pest control, and plant disease [1–4]. A key component of soil biological health is the diversity of soil biota species within function classes performing these ecosystem services. The rates and magnitudes of these ecosystem services are determined by the composition and abundance of the diverse soil biota [5,6]. The assemblages of soil biota found in agricultural soils are known to be sensitive to management practices such as tillage, organic and inorganic amendments, pesticides and herbicides application and can be influenced by crop rotation since soil biota assemblages can be crop-specific [7–10]. It is also now well established that agricultural intensification of soils reduces soil biodiversity [11]. However, there is an intriguing suggestion that soil biota could be manipulated for certain desired outcomes in agricultural management, a process dubbed “soil ecological engineering” [12]. One potential method of soil ecological engineering involves the use of management practices such as mulching and compost application versus synthetic nitrogen application, additives that are known to have a direct impact on soil biota. The biological health of agricultural soils in particular has been shown to increase crop yields through farm compost amendments [13,14].

Soil nematodes are a crucial component of soil biota as they are among the most numerous and diverse organisms found in agricultural soils [15]. Nematodes are present on all trophic levels, ranging from primary consumers to specialist predators [16]. In addition to being ubiquitous, nematodes react measurably to disturbances, and furthermore are easily allocated to trophic groups and identified into functional groups, making them ideal indicators for the soil food web [15,17,18]. Nematode-based weighted indices incorporate functional roles and life history strategies, and as such provide information about the nematode community structure in stressed, enriched, stable, structured and decomposition environments, and provide important information on the dynamics of soil food webs [19–21].

Synthetic fertilizers have been shown to have a large impact on nematode community structure by decreasing fungivorous nematode abundance compared to manure application [22]. In compost-treated soil, total nematode density has been found to increase, with a particular increase of bacterivorous, fungivorous and predacious nematodes [22,23]. However, compost is usually treated as an organic material that benefits the soil, not as a complex matrix with its own biological properties. Thus, the effect of compost is considered indirect. There is a significant gap in the literature on the potential persistence of any nematodes present in the organic amendments after their addition to the soil. Although Steel *et al*. [24,25] revealed high densities of nematodes in compost (between 300–7920 individuals / 100g dry weight of compost) and at the same time an increase in nematode density in soils directly after compost addition, it was not determined if this was either a direct or indirect effect of the compost. Indeed, determining which nematodes originated from the compost after being mixed into the soil is exceedingly difficult using light microscopy. Thus, here we employ metagenomics methods in our experiment to investigate the fate of the nematodes in compost when it is applied to an agricultural soil.

In order to examine both the direct and indirect effects of compost and synthetic nitrogen fertilizer on nematode communities, we sampled a long-term agricultural research site. The aims of this study were: (1) to evaluate the influence of compost amendments and fertilizer additions on the dynamics of free-living and plant-parasitic nematode communities in agricultural soils; (2) determine whether the compost nematodes persist in the soil after application and (3) to compare the suitability of metabarcoding and morphological identifications for ecological studies on soil nematode community dynamics, with regard to their respective workload demands and efficacy.

## Methods

### Field site

**Fig 1:**
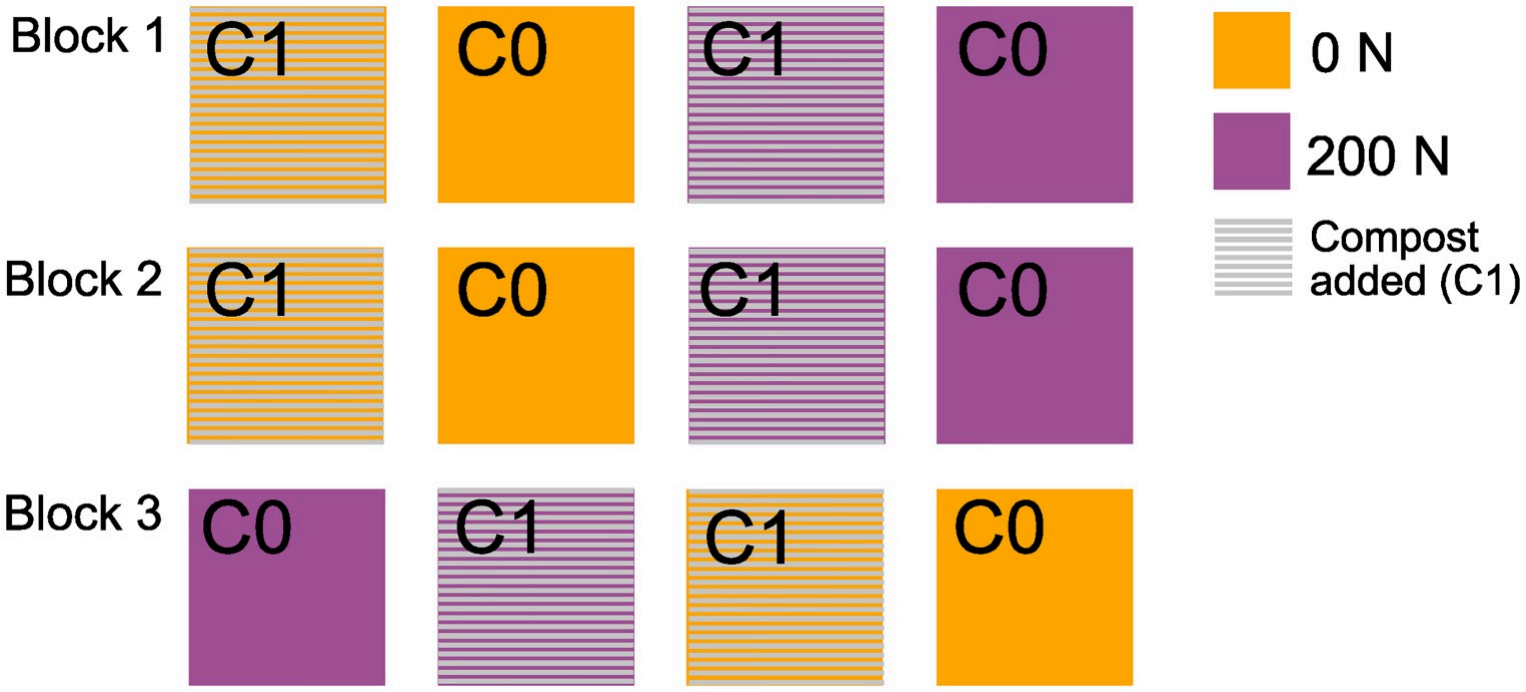
Experimental set up. C1 indicates plots had compost added, while C0 indicates no compost was added. Nitrogen fertilizer was added to plots in purple (200 N), or no nitrogen added was added (orange plots).

A long-term farm compost experiment was initiated in 2004 at Ghent University’s experimental farm in Melle. The farm is situated at 50°59'N, 03°49'E at an elevation of 11m above sea level. The site has a temperate climate with mean annual precipitation of 883 mm and mean annual temperature of 11°C in the period of 2015–2017. A detailed overview of weather data including precipitation, air and soil temperature over each season of sampling is provided in Table 1. The soil is an alfisol with a loamy sand texture comprised of 9% clay, 12% silt, 76% fine sand and 4% coarse sand. The experimental plots were set up using a strip-split plot design [26] with three replicates for each treatment (see Fig 1). Crop was the horizontal factor, nitrogen (N) fertilizer the vertical factor and compost (C) the subplot factor. Nitrogen and compost treatments were fixed, but the crop changed within the plots depending on the prescribed crop rotation. The crops grown were fodder beet (*Beta vulgaris* L. spp. *vulgaris* cv. ‘Rialto’), forage maize (*Zea mays* L. cv LG31.218), Brussels sprouts (*Brassica oleracea* L. cv. ‘Rinus’) and potato (*Solanum tuberosum* L. cv. ‘Challenger’). Every April the trial was ploughed to a depth of 30 cm. Compost or nitrogen fertilizer (calcium ammonium nitrate, 27%N) were spread by hand and rotary-harrowed to a depth of 10–15 cm to incorporate into the soil. Nitrogen treatment plots received 0, 100 or 200 kg N per hectare each year. Compost was applied at a rate of 50m^3^ per hectare each year. Compost was created from approximately 40% green waste materials (straw, grass, hay, crop residue or soiled ensilaged maize) and 60% brown material (woodchips and/or tree bark). Composting was performed in a windrow system on a concrete floor. Weeds were controlled with appropriate herbicides. Fungicides in Brussels sprouts and potato, and insecticides in Brussels sprouts, were applied according to good agricultural practice. Sowing and planting were performed using small agricultural equipment. Further details of the experimental site set up and details of P and K fertilization are described in D’Hose *et al*. [27]; however, only samples from plots treated with 0 or 200kg N ha^-1^ y^-1^ were analyzed in the current paper.

**Table 1:** Average maximum and minimum daily air and soil (5cm depth) temperatures and average, maximum and minimum daily precipitation recorded at the experimental site over the sampling period.

	2015			2016			2017
	Spring	Summer	Fall	Spring	Summer	Fall	Spring
**Average max air temperature (°C)**	16.7 ± 4.8	22.6 ± 4.2	13.7 ± 3.6	16.7 ± 4.8	23.7 ± 3.6	11.7 ± 5.2	18.8 ± 5.8
**Average min air temperature (°C)**	6.2 ± 4.1	12.3 ± 3.0	7.1 ± 3.8	7.6 ± 4.5	13.6 ± 2.4	4.4 ± 4.4	13.0 ± 5.2
**Average soil temperature (°C)**	13.8 ± 4.8	19.7 ± 3.7	9.8 ± 2.9	13.8 ± 4.8	20.6 ± 2.4	7.8 ± 4.9	15.6 ± 6.2
**Average precipitation (mm)**	1.8 ± 3.7	2.5 ± 4.9	2.6 ± 4.4	3.4 ± 7.7	2.7 ± 6.5	1.8 ± 4.7	0.8 ± 2.2
**Minimum precipitation (mm)**	0.0	0.0	0.0	0.0	0.0	0.0	0.0
**Maximum precipitation (mm)**	16.9	23.7	25.1	54.5	41.9	27.9	16.2

Average ± standard deviation. Spring is considered as being from the 20th March– 20th June. Summer from the 21st June– 21st September, Fall from the 22nd September until the 21st December.

### Soil sampling

**Fig 2:**
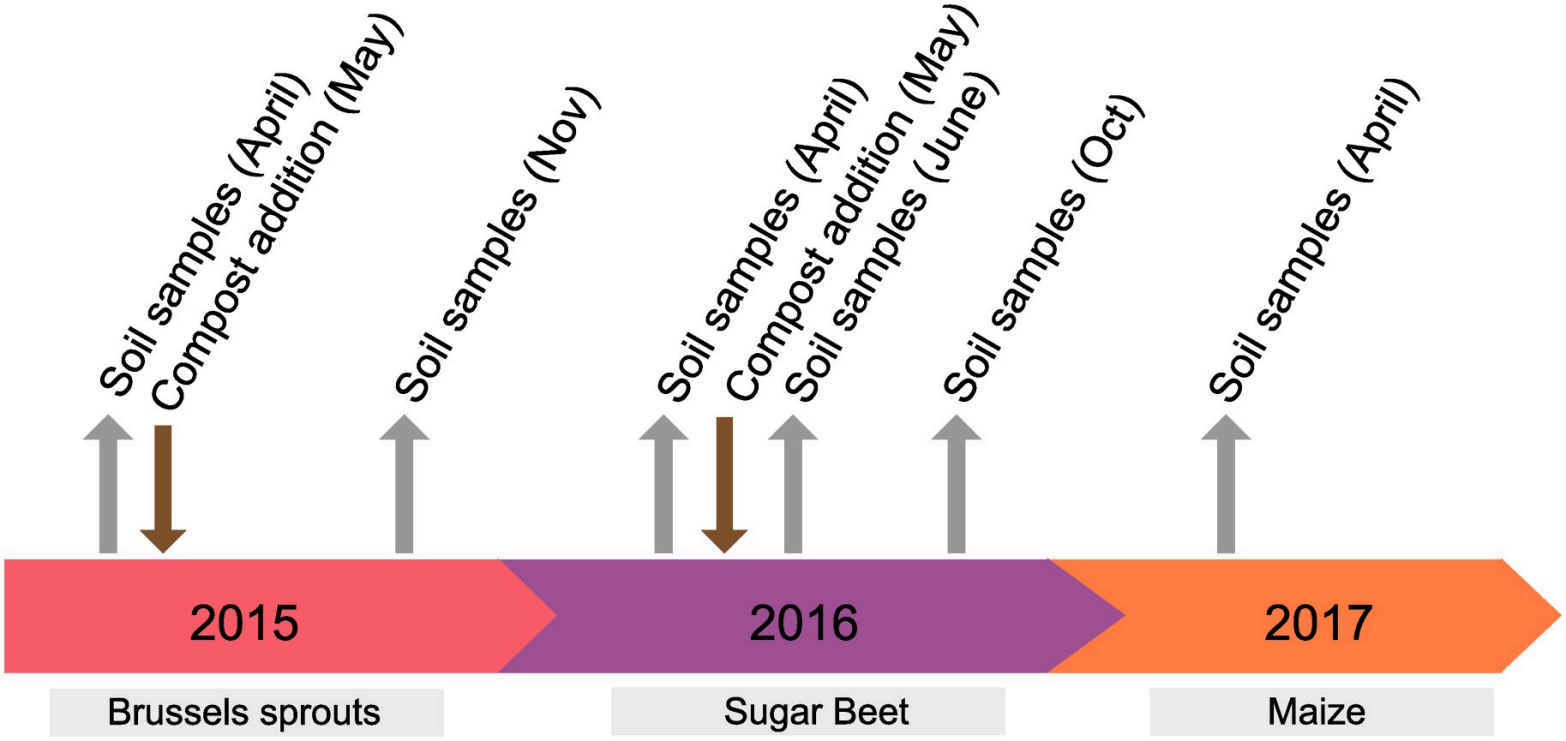
Timeline showing the soil sampling time points and when compost was added to compost plots. Below the crops grown in each year are shown.

From each plot, ten random soil cores of the upper 10cm of soil using a 2.5cm diameter auger were taken. Cores from each plot were bulked and mixed together in a single plastic bag and stored at 4°C until processed. Soil samples were taken on six time points: 29 April 2015, 9 November 2015, 13 April 2016, 16 June 2016, 21 October 2016 and 6 April 2017. These time points were used to reflect the effect of compost and N fertilizer on the soil nematode community and persistence of nematodes from the compost in the soil: preceding compost/N fertilizer application in the spring, several weeks after compost application, and at the end of the growing season. Sampling several weeks after compost application was not possible in 2015 and in 2017 due to practical constraints. The compost and N fertilizer addition took place after the first sampling date of each year (Fig 2). Plots are identified as follows: 200 kg N ha^-1^ y^-1^ only, (C0_200), compost and 200 kg N ha^-1^ y^-1^ (C1_200), compost only (C1_0), no compost and no nitrogen (C0_0).

### Nematode community analysis

To extract the nematodes from the soil samples, 300 ml of soil was placed on a modified Baermann tray for 48 h, and the nematodes were subsequently retrieved using a 38-μm sieve [28], and enumerated using a Leica stereomicroscope. Two extractions were done for each plot at each sample point; one to be used for morphological identification and one for metabarcoding analysis. For morphological identification, 4% paraformaldehyde was heated to 70°C and an excess (4–5 mL) was quickly added to the specimens to kill and preserve them [29]. The preserved nematodes were stored at 4°C until further processing. Samples to be used for metabarcoding analysis were frozen in water at -80°C.

For morphological identification, the nematodes were processed to glycerol following the glycerol-ethanol method [29,30] and mounted on slides. For each sample, at least 100 (both adult and immature) nematodes were identified to genus or family level with a Zeiss Axioplan 2 compound microscope.

The compost nematode community was analyzed by extracting nematodes from three replicates of 300 mL of compost using a modified Baermann tray. After 48 hours, the nematodes were retrieved, enumerated with a stereo microscope (Leica Mz 95) and approximately 100 nematodes were mounted on slides as described above. A compound microscope (Zeiss Axioplan 2) was used to identify nematodes in order to calculate the Nematode Index of Compost Maturity. The Nematode Index of Compost Maturity (NICM), based on nematode abundance, fungal feeding / (fungal feeding + bacterial feeding) nematode ratio, presence of diplogasterids and presence of more than one fungal-feeding taxon [25] was calculated to assess the biological maturity of the compost. Only compost applied in June 2015 was assessed using this method due to missing samples from the 2016 compost application.

Molecular characterisation of the nematode community was carried out following the protocol described in Waeyenberge *et al*. [31]. Briefly, metabarcoding analysis was carried out by extracting DNA using the DNeasy Blood and Tissue Kit (Qiagen). Amplicon libraries of approximately 490bp were produced with the Illumina Nextera XT Indext Kit v2 using 18S rRNA gene primers (NemFopt and 18Sr2bRopt) and sequenced with Illumina MiSeq v3 (2x300 bp) as described in Waeyenberge *et al*. [31]. The raw reads were uploaded to the Sequence Read Archive (SRA) of NCBI under BioProject PRJNA607002. Primers were removed from the resulting sequences using Trimmomatic v0.32 [32], forward and reverse reads were merged using PEAR v0.9.8 [33] and resulting sequences with more than 6 expected errors or more than 0 N’s were removed using vsearch fastx_filter v2.6.0 [34]. Amplicon sequence variants (ASVs) were derived from the sequences using DADA2 [35] in R v3.4.3 (R core Team, 2018). These sequences were compared to a curated nematode sequence database, derived from SILVA128, for taxonomic assignment across multiple ranks using the naïve Bayesian classifier method with a minimum bootstrap support of 80. When molecular identification was unclear, sequence data were blasted in GenBank and manually compared with similar sequences or, if needed, analyzed in a phylogenetic tree with related sequences. This resulted either in a more precise match or assignation to a higher taxonomic rank (i.e. family level instead of genus level). ASVs with less than 30 read counts across all samples were removed prior to analysis. All counts for ASVs matching to the same genus were summed, and any non-nematode taxa still present were also removed. Four samples with less than 300 read counts were considered failed and removed from further analysis (samples from October 2016: nitrogen only, compost and nitrogen, compost only, and one compost replicate from 2015). The final average read count of the remaining samples was 30,094 ± 9690 read counts per sample. To follow compost nematodes from compost into the soil, only metabarcoding data were used, so individual ASVs with unique sequences could be tracked, and only ASVs with more than 30 read counts over the 5 compost samples were considered.

The nematode community was represented using relative and absolute abundances (or read counts in the case of metabarcoding data) of nematode trophic groups and taxa richness. A total of five trophic groups were used: bacterivorous, fungivorous, herbivorous, omnivorous and predaceous. A c-p value [19] was assigned to each nematode taxon, and the Maturity Index was calculated with the online web application NINJA (https://sieriebriennikov.shinyapps.io/ninja; [19, 36]).

### Data analyses

All data were analyzed with R statistical software (version 3.5.1) using Rstudio (Version 1.1.383). The differences between the compost-treated and nitrogen fertilizer treatment nematode communities over the different sampling dates were tested using PERMANOVAs employing abundance data for morphological data and read counts for the metabarcoding data with Bray-Curtis dissimilarity matrices and the R-package Vegan 2.5–3 [37]. The influence on the indices and the nematode feeding types between treatments were tested using a one-way ANOVA. Differences between nematode assemblages in the different samples were visualized with non-metric MultiDimensional Scaling (nMDS) using the R packages phyloseq [38], ggplot2 [39] and RcolorBrewer [40].

## Results

### The influence of nitrogen fertilizer and compost on the nematode community

**Fig 3:**
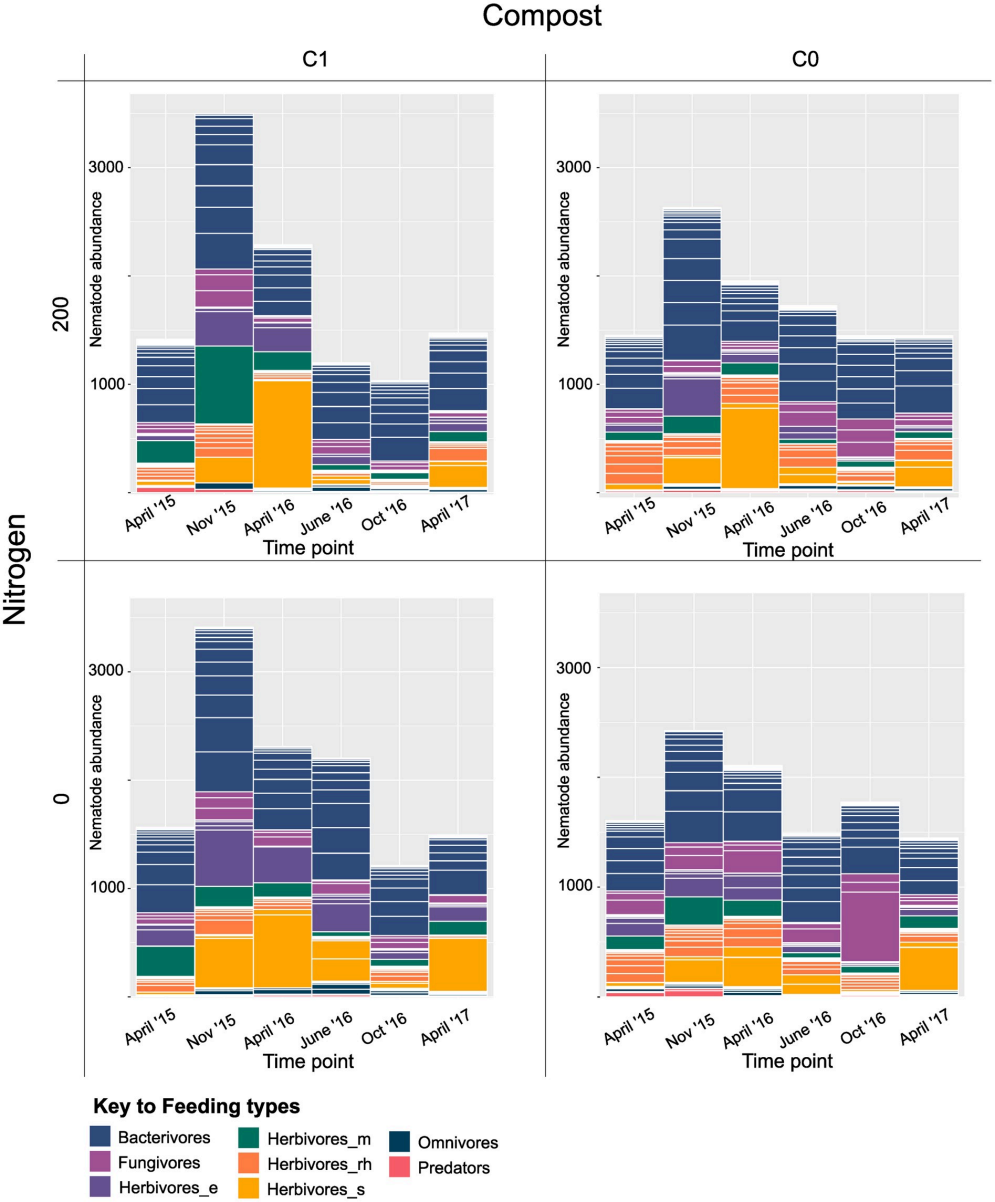
Change in abundance of nematodes in 300 ml of soil of treatment plots over time based on morphological data. Each bar represents the average of three replicate samples. Nematodes are classified by feeding types: bacterivores, fungivores, ectoparasitic herbivores (herbivores_e), migratory herbivores (herbivores_m), epidermal/root hair feeders (herbivores_rh), sedentary parasites (herbivores_s), omnivores and predators. Substacks indicate different taxa.

**Fig 4:**
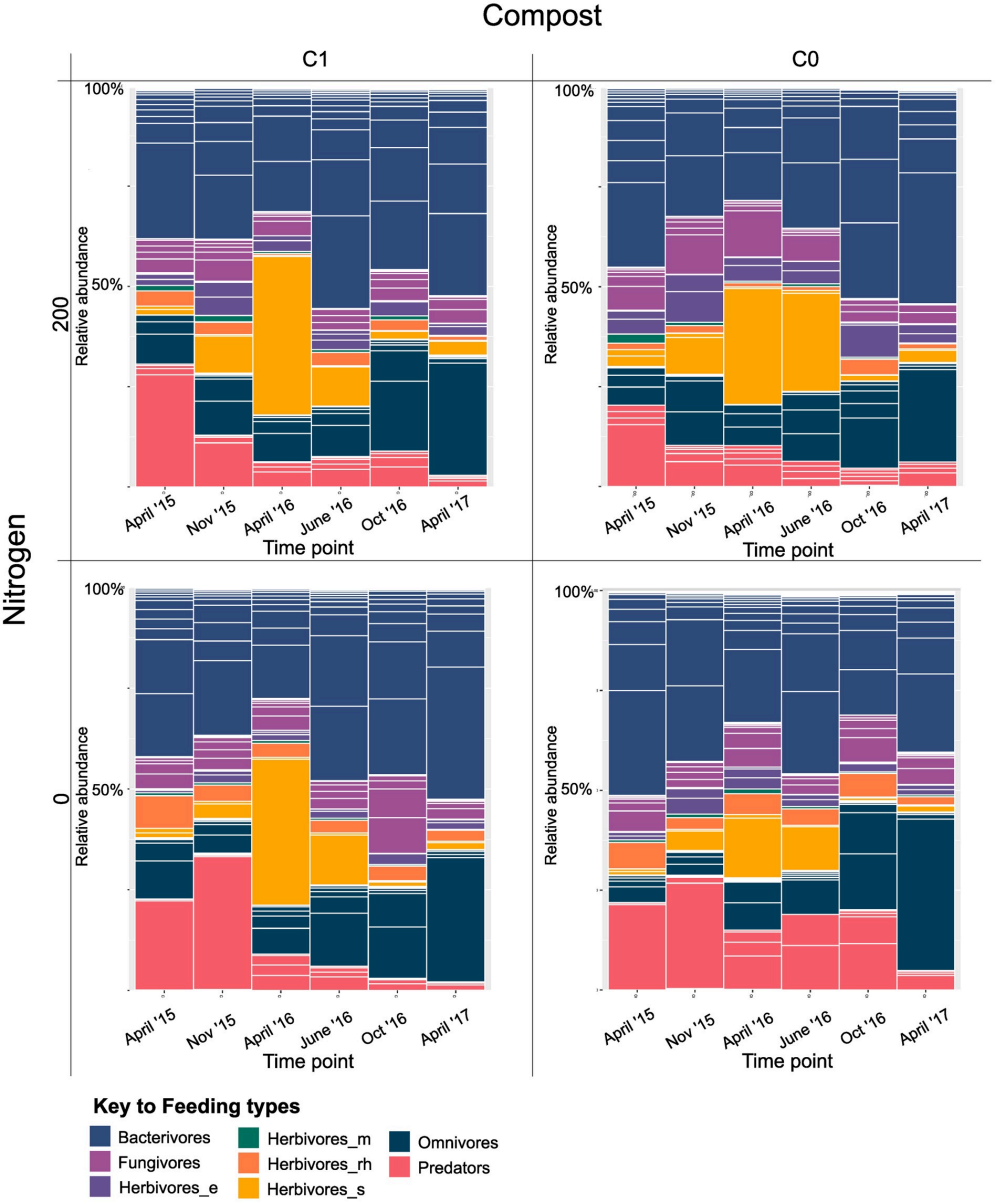
Change in relative abundance of nematodes in treatment plots based on read counts of metabarcoding data from 300 ml of soil, showing the shift of relative nematode abundance and feeding type over time. Each bar represents the average of three replicate samples. Nematodes are classified by feeding types: bacterivores, fungivores, ectoparasite herbivores (herbivores_e), migratory herbivores (herbivores_m), epidermal/root hair feeders (herbivores_rh), sedentary parasites (herbivores_s), omnivores and predators. Substacks indicate different taxa.

Based on the Bray-Curtis resemblance matrix, the nematode community in soils was significantly affected by nitrogen addition in both metabarcoding (PERMANOVA analysis, P<0.05, df = 1) and morphological data (p<0.01, df = 1), while compost addition did not provoke any significant effects according to either morphological or metabarcoding data (PERMANOVA analysis p>0.2, df = 1). Nematode feeding types were highly variable through the seasons (Fig 3). For all samples, the greatest abundances were found in November 2015. Bacterivorous nematodes were nearly always the dominant group, except in April 2016 where sedentary herbivorous nematodes prevailed in all treatments (with the exception of the treatment with no inputs) and October 2016 when fungivorous nematodes were the most abundant (in treatment with no inputs). Heatmaps of taxa abundance are shown in the supplementary information (S1–S4 Figs). The fluctuation of nematode feeding types is also shown in the metabarcoding data (Fig 4), with a similar dominance of bacterivorous nematodes at most timepoints and an increase in sedentary herbivorous nematodes in April 2016. Over all timepoints, there was a small but significant (p<0.05) negative effect of nitrogen fertilizer addition on fungal feeding nematodes (Table 2, p = 0.01), predators (Table 2, p = 0.04) and a positive effect on herbivores (Table 2, p = 0.02). However, these significant effects were only observed in the metabarcoding results. Other, noticeable differences between metabarcoding and morphological identification include the proportion of omnivorous and predaceous nematodes which have a larger presence in the metabarcoding data compared with the morphological data. The nematode density (based on morphological identification only) was only significantly influenced by the factor timepoint (one-way ANOVA, p<0.001), while N fertilizer or compost, did not appear to have an effect on density. (Table 2).

**Table 2:** One-way ANOVA results for soil nematode abundance and trophic groups between compost, nitrogen addition, treatment, and timepoint using morphological and metabarcoding data.

	Compost addition		Nitrogen addition		Treatment		Timepoint	
	F-test	P-value	F-test	P-value	F-test	P-value	F-test	P-value
**Total density**								
*morphological*	0.0113	0.9155	0.4788	0.4913	0.2052	0.6520	3.4416	<0.001
**Trophic group**								
**Fungivore**								
*morphological*	1.188	0.338	3.227	**0.011**	1.486	0.143	1.637	0.053
*metabarcoding*	1.207	0.276	0.274	0.603	0.58	0.631	3.195	0.012
**Bacterivores**								
*morphological*	0.609	0.809	1.750	0.069	0.795	0.794	3.574	**<0.001**
*metabarcoding*	0.204	0.653	0.0002	0.989	0.069	0.976	5.3	**<0.001**
**Herbivores**								
*morphological*	0.777	0.593	2.613	**0.023**	1.280	0.198	4.454	**<0.001**
*metabarcoding*	0.207	0.651	2.238	0.139	0.935	0.429	23.585	**<0.001**
**Omnivores**								
*morphological*	1.200	0.303	1.773	0.136	1.153	0.331	1.865	0.018
*metabarcoding*	0.154	0.696	0.063	0.803	0.149	0.93	9.901	**<0.001**
**Predators**								
*morphological*	1.024	0.44	2.358	**0.04**	1.842	**0.03**	1.581	0.061
*metabarcoding*	0.452	0.504	0.8634	0.3561	1.05	0.376	26.906	**<0.001**

Morphological results are based on abundance data, metabarcoding data is based on relative abundance of feeding types.

**Fig 5:**
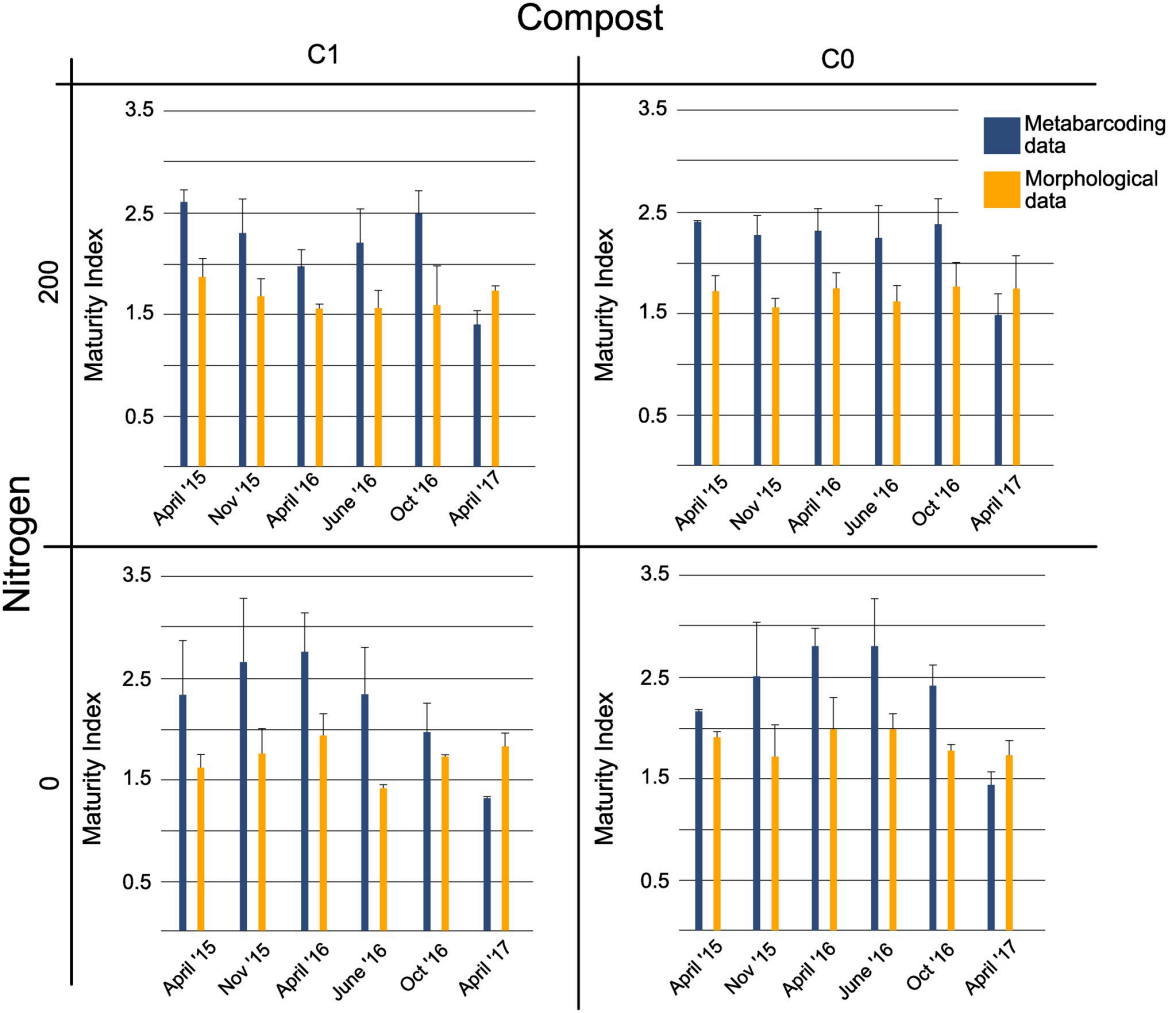
Maturity index of the nematode community using morphological and metabarcoding data for each treatment and timepoint. Each bar represents the average of three replicate samples, error bars indicate the standard deviation.

The Maturity Index (MI), calculated from both morphological and metabarcoding data, did not vary significantly between treatments and timepoints (Fig 5). The average MI over all treatments based on metabarcoding data was significantly higher than the MI based on morphological data (ANOVA, df = 1, F-value = 30, p<0.0001). The standard deviations when averaged over time were also on average 3.3 times higher for the metabarcoding data, showing more variation in the MI for the metabarcoding data than the morphological identifications.

### Compost nematode persistence after application of compost

**Fig 6:**
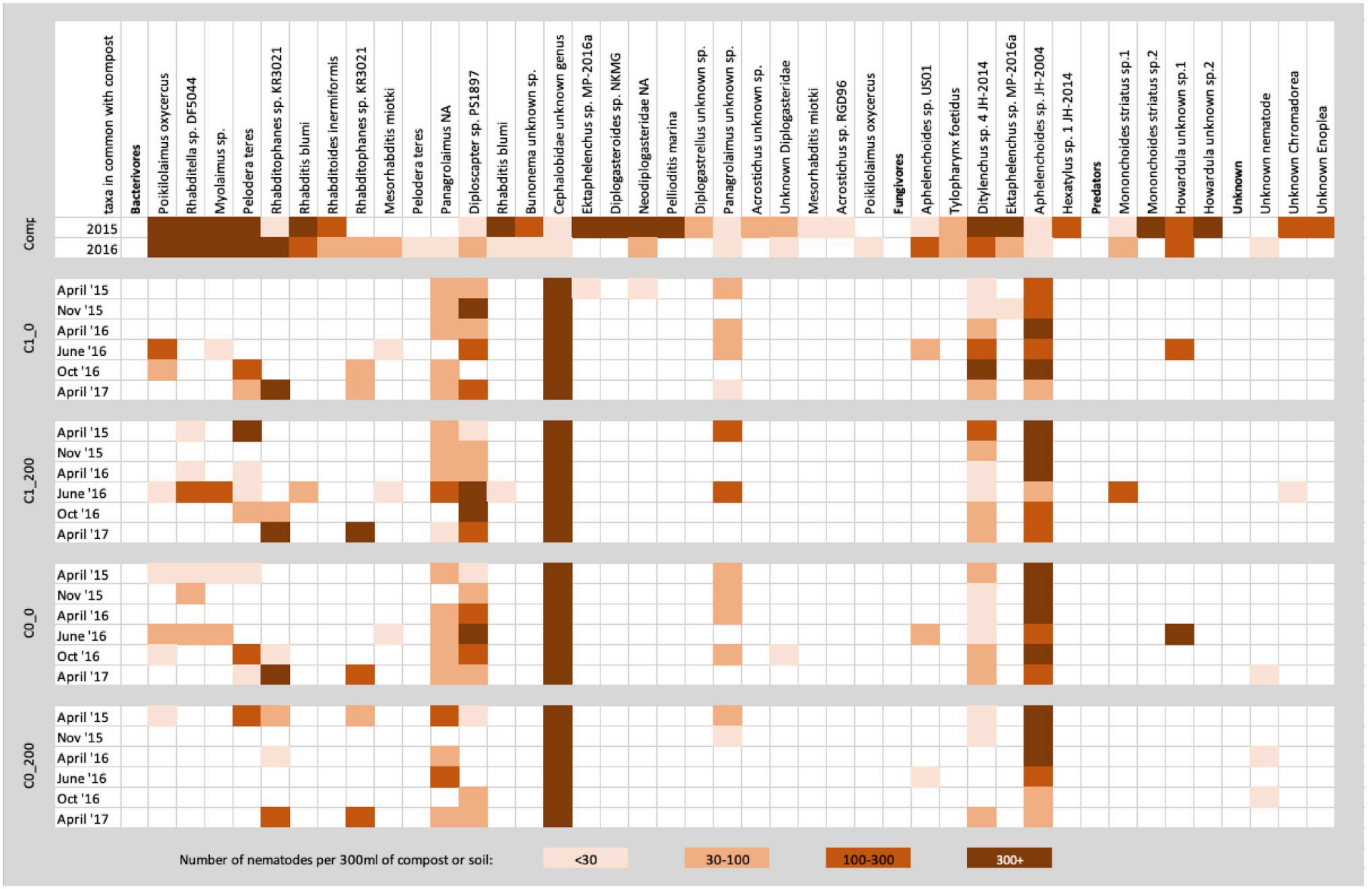
Heat map of average read counts of taxa present in 300 ml of compost (presence only counted if total read count was above 30 over all 5 compost samples), and soil nematodes in common with compost-present taxa.

The NICM of the compost from June 2015 was calculated as 2.06 +/- 0.01. This was calculated with morphological data since NICM requires absolute abundance data. However, in order to follow the persistence of individual taxa from the compost into soil, only species-specific metabarcoding data have been used, i.e. unique compost nematode sequences. Fifteen families, twenty-two genera and thirty-nine ASVs in the metabarcoding analysis were recovered from the compost samples, before their addition to the soil. The two composts (April 2015 and April 2016) had different nematode community compositions (Fig 6). The taxonomic assignments of the ASVs showed that the April 2015 compost was dominated by *Ektaphelenchus* sp. MP-2016a, *Diplogasteroides* sp. NKMG, *Rhabditis blumi and Rhabditella* sp. DF5044. Compost from April 2016 was dominated by *Poikilolaimus oxycercus*, *Rhabditella* sp. DF5044, *Myolaimus* sp. and *Pelodera teres*. Fourteen ASVs were unique to the compost samples, while twenty- four nematode ASVs present in the composts were also present in soils. Remarkably, most compost-soil-shared ASVs were also present in soils where no compost had been added, i. e. no clear difference between the presence of “compost nematodes” in compost-amended soils vs reference soils was observed (Fig 6). Only four nematode compost-nematode ASVs were present only in the compost-amended soils: *Ektaphelenchus* sp. MP-2016a, *Rhabditis blumi*, and two unidentified species of Diplogastridae and Chromadorea. They were only present in the soil at very low abundances (less than 30 read counts) and were not detected at the next sampling.

### Comparison of morphological and metabarcoding identification

**Fig 7:**
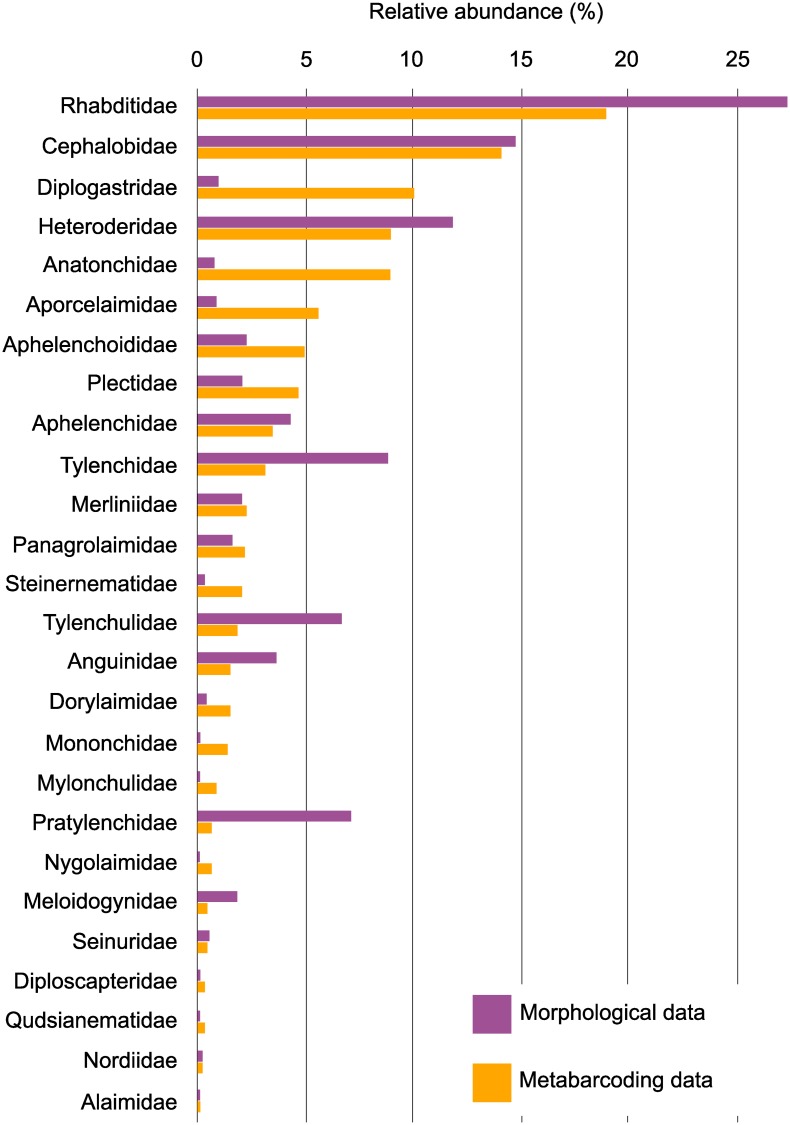
Relative abundance of the nematode community at the Family level, over all time points and treatments of nematode communities using morphological and metagenomic identification methods. Only families found in both identification methods are shown and with greater than 0.2% relative abundance.

Overall, metabarcoding analysis identified more taxa than the morphological analysis; 42 families and 62 genera *vs* 30 families and 55 genera, respectively. Furthermore, distinct differences in the relative abundance of several groups were observed over all timepoints (Fig 7). Certain groups appeared highly over-represented in the metabarcoding analysis when compared with the morphological identification, especially Aporcelaimidae, Anatonchidae and Diplogastridae. Conversely, Pratylenchidae, Rhabditidae and Tylenchidae were underrepresented.

**Fig 8:**
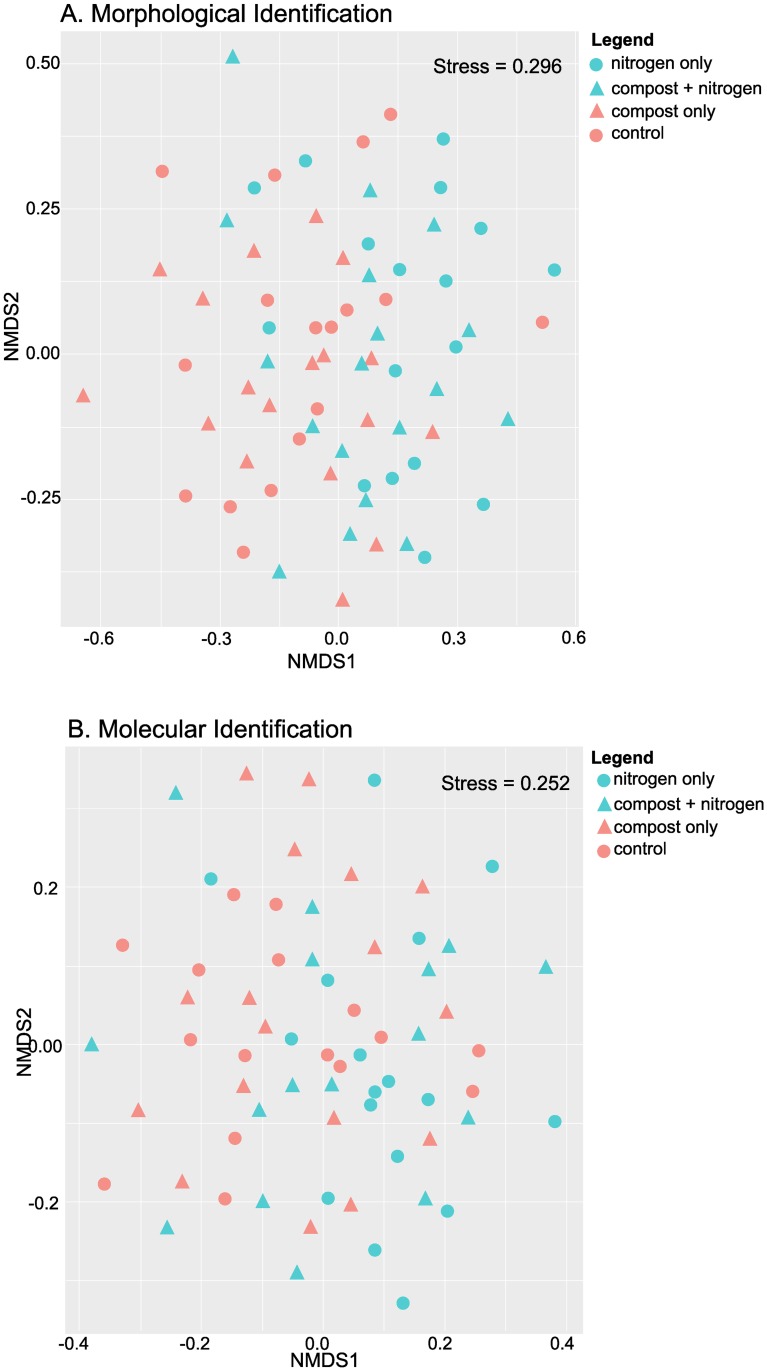
Bray-Curtis based non-metric multi-dimensional scaling (NMDS) plots of all samples in either morphological identified samples (A) or molecular identified samples (B).

Non-metric multi-dimensional scaling (NMDS) plots with Bray-Curtis dissimilarity were constructed to represent the nematode community on a non-metric scale. While in neither the metabarcoding nor morphologically analyzed samples was any strong grouping visible, there was a difference (PERMANOVA analysis, p <0.05, df = 1) between the two nitrogen treatment groups (N 200 vs N 0; Fig 8). No clear distinctions were noticed between the compost and no compost treatments.

Similarly, as noted above, while the metabarcoding-based Maturity Index is higher, when the MI of plots averaged over time are ranked, a similar pattern is found between the two data sets. In both metabarcoding and morphological data, treatment with no inputs had the highest overall average MI, followed by nitrogen only treatment, compost and nitrogen treatment, and finally the lowest MI was found in compost only treatment.

## Discussion

Soil nematode communities are shaped by several environmental and agricultural management factors. Here we investigated the influence of nitrogen and compost application over the course of several growing seasons, paying special attention to the potential impact and peristence of nematodes originating from compost. Results were obtained and compared using two identification methods; classical morphological identification and metabarcoding.

The significant differences between the nematode communities at different timepoints were expected, as many nematodes are known to have seasonal variations. For example, the observed peak of *Heterodera* in our samples in April 2016 and April 2017 is similar to the study of Verschoor *et al*. [42] who observed a peak of *Heterodera* in the spring. Several other plant-parasitic nematodes were found to have their lowest densities in the winter and early spring. Another study found that plant-parasitic nematode populations decreased by the end of the crop cycle, increasing again when the growing season recommenced, and peak bacterivore populations in the middle of the rainy season [41]. In addition, we extracted nematodes from soil and not from plant roots which may harbour considerable numbers of certain developmental stages of endoparasitic nematodes, depending on the plant host status, plant maturity and life cycle of the nematode. However, annual population cycles may not be as noticeable for nematodes which have short life cycles and can produce several generations in a year such as Tylenchidae, Aphelenchidae, Rhabditidae [42]. Certain genera can also have individual responses to the soil moisture conditions: *Acrobeles* and *Pratylenchus* were found in high abundances in wet soils, *Cephalobus* and *Meloidogyne* could persist in dry soils, while the genus *Helicotylenchus* is able to thrive in a large range of soil moisture [41]. The overall highest nematode abundances were observed in the autumn (November 2015). However, samples from autumn 2016 had low abundances compared with the previous year, possibly due to lower temperatures and reduced rainfall.

As well as climatic conditions, soil inputs are also capable of influencing the nematode community. In line with previous findings [17,43,44], nitrogen fertilizer application showed small but significant effects on the soil nematode community. A significant increase in overall nematode abundances was not observed with nitrogen fertilizer application, although a decrease in fungivorous nematodes, and an increase in plant-parasitic nematodes was displayed in the nitrogen-only treatments. Nitrogen fertilizers are reported to increase bacterivorous nematode abundances and suppress fungivorous and omnivorous nematodes [44]. Bacterivorous nematode abundance increases are thought to occur due to increased nutrient availability when N fertilizer is applied. Our results do not support the results of Li *et al*. [45], whose study found nitrogen fertilizers to increase fungivorous and plant-parasitic nematode populations while decreasing bacterivorous nematodes.

Remarkably, we did not find compost to exert any noticeable effects on the soil nematode community. Compost effects on soil nematode communities are known to be variable, with several studies showing clear effects [24,46,47], while one study also reported no noticeable effects following the addition of compost [48]. Our compost was shown to have a Nematode Index of Compost Maturity (NICM) of only 2.06, which, being lower than 3, is considered a low biological compost maturity [24]. This may explain the negligible effect on the soil nematode community. However, research using NICM as a measure of compost biological quality is still in its infancy and the exact relation of NICM and its effect on soil nematode communities remains to be investigated.

Regular tillage may also have masked any effect of compost application by regularly disturbing the soil food web. Tillage is well known to have a significant impact on the nematode community, including the MI [9,47,49]. The lack of cover crops in between growing seasons on the sampled plots may also have reduced the resilience of the soil food web and the associated soil nematode community. Cover crops provide a continuous food source for the soil food web, which is reflected by higher nematode abundances and increased nematodes of a higher trophic guild in a soil [9,50]. Thus, with tillage and missing cover crops, the soil nematode community is not able to mature from a state of enrichment, as seen in the MI values which are similar throughout the different treatments. This suggests that the soil biota is influenced by multiple management practices which may have stronger effects than the application of compost, most likely leading to no significant nematode community changes following compost application.

As mentioned above, several studies have investigated the effect of different composts or organic amendments on soil nematode communities, but so far no attempt has been made to directly follow the development of nematodes originating from the amendments after their application to soil [22,46,47,51,52]. Nematodes are present in very high numbers in composts, and occur in distinct nematode communities that can be used to determine the biological maturity of a compost [24,25,53]. It was hypothesized by Steel *et al*. [24] that the increase of certain taxa after compost amendment may at least partly be a direct effect of inoculation of compost nematodes, and that persisting predators may offer biocontrol potential [18,54]. In our study, metabarcoding has been used to trace, for the first time, unique compost nematode sequences in the soil after compost application. However, our results also revealed several “compost nematodes” in soils that were not treated with compost. The presence of “compost nematodes” in both compost and soil indicates the ubiquity of opportunistic microscopic animals, such as rhadiditid, cephalobid and aphelenchid nematodes. Only four compost nematodes (*Ektaphelenchus* sp. *MP-2016a*, *Rhabditis blumi*, and two species of Diplogastridae and Chromadorea) were retrieved solely in compost-amended plots. The application effect of introducing compost nematodes appears to be only temporary since they were present in very low numbers in the soil and were not detected more than 6 months after compost application. This indicates that inoculation of the soil with compost nematodes is not straight forward and confirms that the free-living nematode community is massively shaped by its environment [15,55]. Steel *et al*. [24] forwarded the fungal-feeding *Ditylenchus* as a plausible candidate to persist in the soil after compost application. However, as this genus is more abundant in biologically more mature composts (with higher fungivorous to bacterivorous nematode ratios), its potential persistence could not be investigated in this study [25].

Metabarcoding of soil nematode communities has the potential to provide fast and increased taxonomic resolution compared with morphological identification [56]. In a similar comparison of morphological and metabarcoding approaches to our study, Plectidae and Longidoridae were found to be overrepresented, while the remaining families were underrepresented [57]. Our results confirmed the over representation of Plectidae based on metabarcoding, but also showed the overrepresentation of several other families, most notably (Mononchidae, Anatonchidae, Aporcelaimidae and Diplogasteridae). On the other hand, several nematode families appeared to be underrepresented in the metabarcoding data as compared to the morphological data, such as Tylenchidae, Pratylenchidae and Rhabditidae. It is likely that the read counts in the metabarcoding data are skewed by body size, such as is seen with the Mononchidae, Anatonchidae or Aporcelaimidae, or by DNA extraction being more successful from certain nematodes than others [31,57,58].

Indeed, several steps in the metabarcoding procedure are prone to potential errors: during DNA extraction and isolation, DNA amplification is limited by the primers selected and the amplification efficiency can be species-specific [59]. These errors may mask or introduce variation which can affect community analyses [57]. There are also limitations in the accuracy of reference sequences databases. Furthermore, and most importantly, metabarcoding is unable to provide absolute quantitative data on the nematode community [60]. The use of a correction factor has been suggested as a method to estimate abundances from metabarcoding data [31], but this still requires further standardization and investigation to determine the classification level at which the correction factor can be applied. Nevertheless, despite the significant methodological differences between morphological and metabarcoding methods actual differences in the interpretation of the results are limited. Some significant effects of nitrogen fertiliser on nematode trophic groups were only revealed based on the morphological method. This may be attributed to the observed under or over-representation of certain taxa. However, given the overall congruence of our major results, i.e. that they are independent of the method used, metabarcoding can be an efficient method for ecological studies such as this one.

## Conclusion

In this long-term experiment, nitrogen application appears to have had significant effects on the soil nematode community, while compost addition did not. Although there were differences between the identification data collected through morphological and metabarcoding methods, the overall patterns indicated for the nematode communities remained the same. Using metabarcoding methods, we found that, contrary to our hypothesis, nematodes from compost did not persist in soil over time.

The effects of both high and low NICM compost on the soil nematode community, and the effect of compost on more intact soil systems (minimal tillage and other management practices that foster soil food web connections, for example cover crops) still remain to be investigated. It is clear that the metabarcoding methods used in this research would be well-suited to do this manner of study.

## Supporting information

S1 Fig Abundance heat maps using morphological identification data for the treatment with compost, no nitrogen fertilizer (C1_0). Lightest shade is 1–25 nematodes per 300 ml of soil, medium shade is 26–100 nematodes per 300 ml of soil and darkest shade is 100+ nematodes per 300 ml of soil.
(TIF)

S2 Fig Abundance heat map using morphological identification data for treatment with no compost, no nitrogen fertilizer (C0_0). Lightest shade is 1–25 nematodes per 300 ml of soil, medium shade is 26–100 nematodes per 300ml of soil and darkest shade is 100+ nematodes per 300 ml of soil.
(TIF)

S3 Fig Abundance heat map using morphological identification data for treatment with no compost, with nitrogen fertilizer (C0_200). Lightest shade is 1–25 nematodes per 300 ml of soil, medium shade is 26–100 nematodes per 300ml of soil and darkest shade is 100+ nematodes per 300 ml of soil.
(TIF)

S4 Fig Abundance heat map using morphological identification data for treatment with compost and nitrogen fertilizer (C1_200). Lightest shade is 1–25 nematodes per 300ml of soil, medium shade is 26–100 nematodes per 300ml of soil and darkest shade is 100+ nematodes per 300ml of soil.
(TIF)

S1 Data (CSV)

S2 Data (CSV)
