# Supplementary figures and images for: Effects of synthetic fertilizer and farm compost on soil nematode community in long-term crop rotation plots: A morphological and metabarcoding approach
Source: PLoS One. 2020 Mar 17;15(3):e0230153. doi: 10.1371/journal.pone.0230153 (PMC7077811; doi:10.1371/journal.pone.0230153)

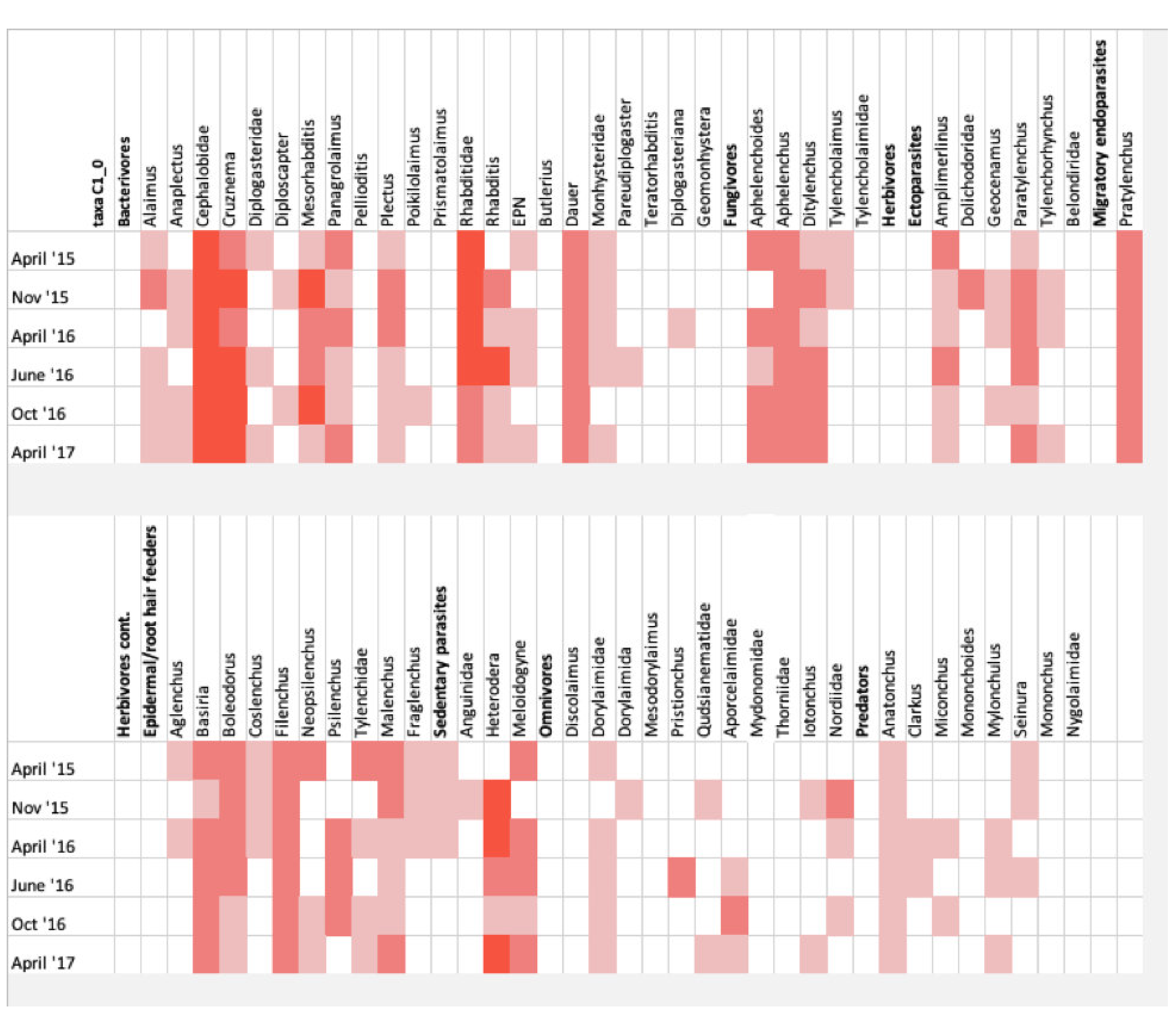

Supplement: S1 Fig — Lightest shade is 1–25 nematodes per 300 ml of soil, medium shade is 26–100 nematodes per 300 ml of soil and darkest shade is 100+ nematodes per 300 ml of soil. (TIF) [file pone.0230153.s001.tif]

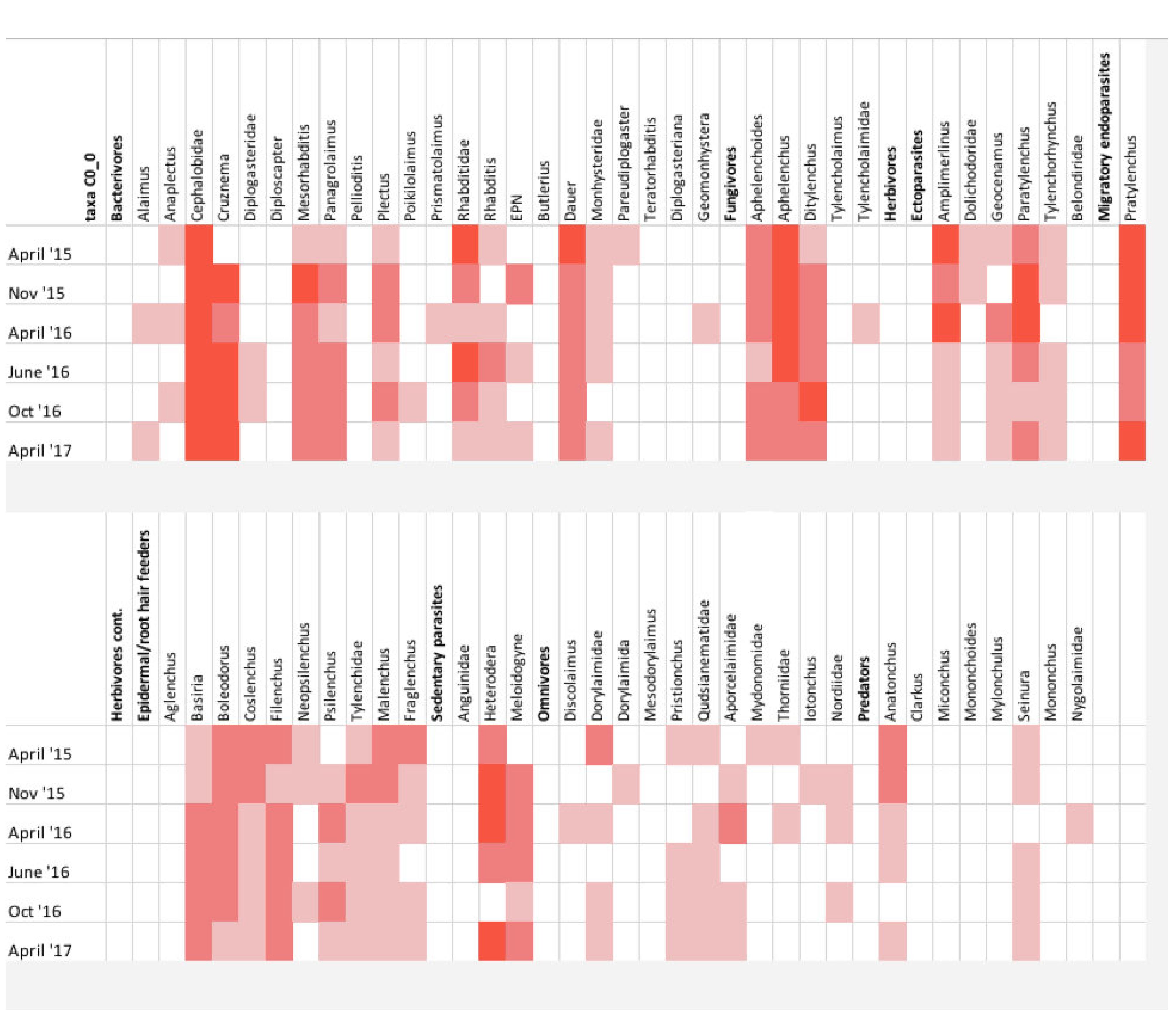

Supplement: S2 Fig — Lightest shade is 1–25 nematodes per 300 ml of soil, medium shade is 26–100 nematodes per 300ml of soil and darkest shade is 100+ nematodes per 300 ml of soil. (TIF) [file pone.0230153.s002.tif]

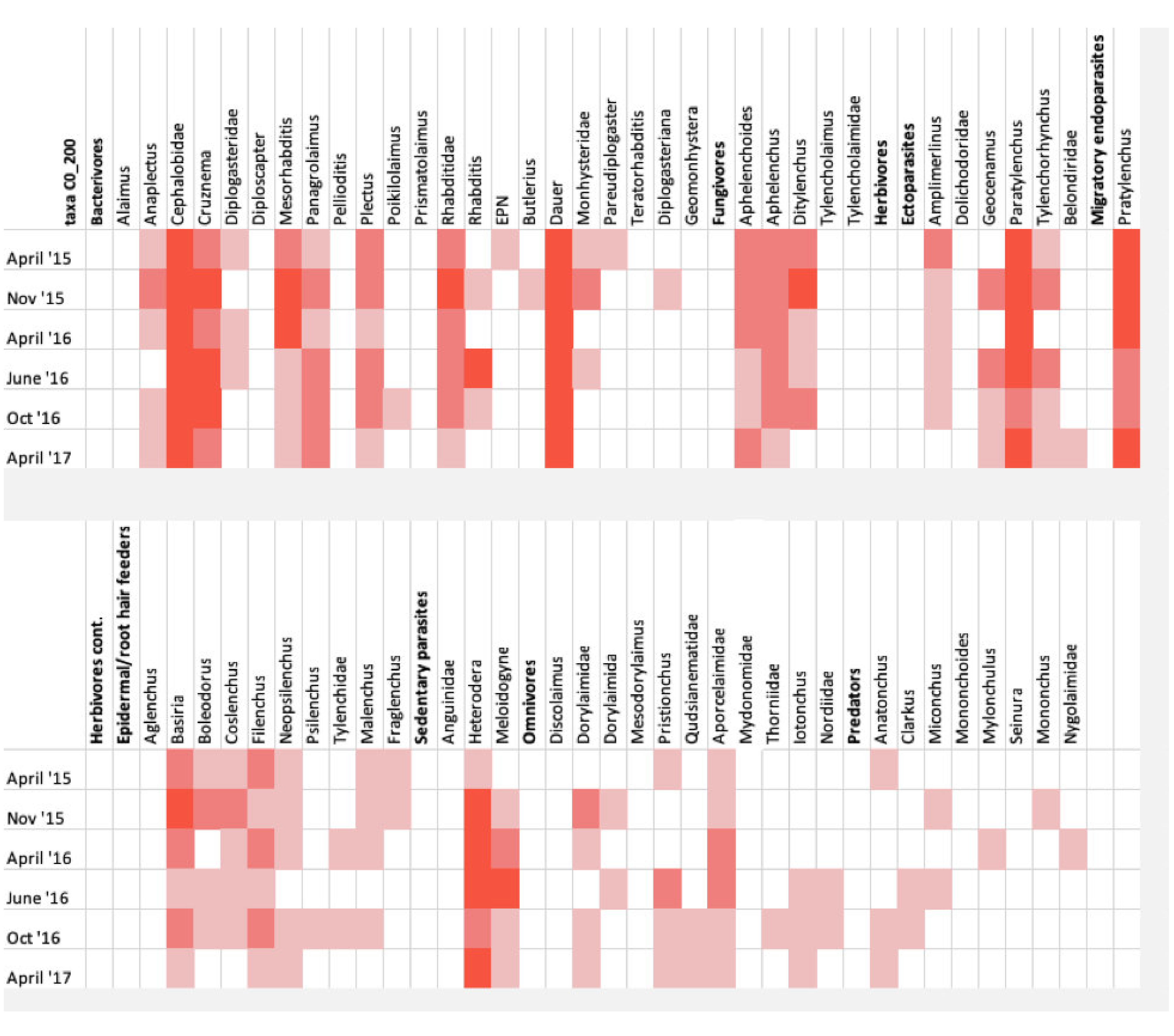

Supplement: S3 Fig — Lightest shade is 1–25 nematodes per 300 ml of soil, medium shade is 26–100 nematodes per 300ml of soil and darkest shade is 100+ nematodes per 300 ml of soil. (TIF) [file pone.0230153.s003.tif]

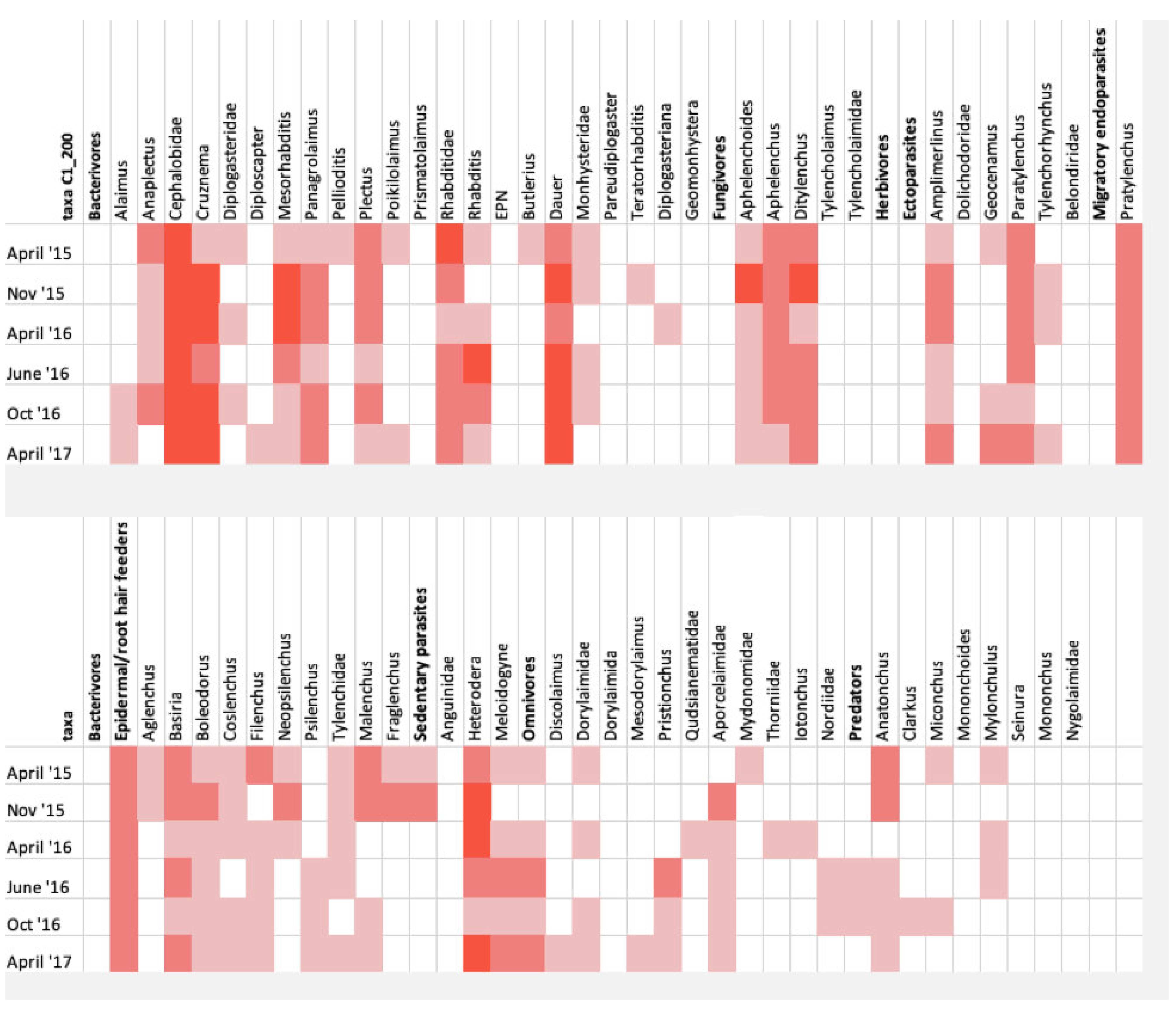

Supplement: S4 Fig — Lightest shade is 1–25 nematodes per 300ml of soil, medium shade is 26–100 nematodes per 300ml of soil and darkest shade is 100+ nematodes per 300ml of soil. (TIF) [file pone.0230153.s004.tif]
